# Iron and Magnesium Co-substituted Hydroxyapatite Nanoparticles in Orthodontic Composite: A Preliminary Assessment

**DOI:** 10.7759/cureus.56388

**Published:** 2024-03-18

**Authors:** Shweta Nagesh, Kirthick Kumaran, Pugazh Mani

**Affiliations:** 1 Orthodontics and Dentofacial Orthopedics, Saveetha Dental College and Hospitals, Saveetha Institute of Medical and Technical Sciences, Saveetha University, Chennai, IND; 2 Dentistry, Saveetha Dental College and Hospitals, Saveetha Institute of Medical and Technical Sciences, Saveetha University, Chennai, IND

**Keywords:** antimicrobial, magnesium, iron, orthodontic composite, hydroxyapatite nanoparticles, dental

## Abstract

Aim

The study aims to characterize Fe and Mg co-substituted hydroxyapatite nanoparticles (FeMgHAPn) and assess the antimicrobial properties of FeMgHAPn-incorporated orthodontic composite.

Materials and methods

FeMgHAPn was synthesized using the sol-gel method, and the prepared nanoparticle powder was characterized using Fourier Transform Infrared Spectroscopy (FTIR), energy-dispersive X-ray analysis (EDX)) and scanning electron microscopic (SEM) analysis. The FeMgHAPn was incorporated into a commercially available orthodontic composite in two concentrations (40 and 20 μL), and the structure was examined using SEM. The FeMgHAPn-incorporated composite was tested for its antimicrobial efficacy against *Streptococcus mutans*, *Staphylococcus aureus*, and *Escherichia coli* using the agar-well diffusion method. The zones of inhibition (ZOI) were measured in millimeters (mm).

Results

The characterization of the FeMgHAPn indicated the successful formation of the nanoparticle without any impurities or byproducts. The high concentration (40 μL) of FeMgHAPn-incorporated orthodontic composite showed the maximum ZOI against all three microbes, followed by the low concentration (20 μL) and the control group.

Conclusion

The FeMgHAPn-incorporated orthodontic composite showed promising antimicrobial activity against caries-causing *S. mutans*, *S. aureus*, and *E. coli*.

## Introduction

Decalcification of the enamel manifesting as a white spot lesion (WSL) is a prevalent unintended consequence of orthodontic treatment [1]. In some instances, the WSLs can be reversed; salivary proteins that remineralize the enamel surface may partially mitigate the chalky appearance. On the other hand, these enamel lesions progress and become irreversible during orthodontic treatment, resulting in carious processes [2]. Based on the available evidence, patients who have WSLs post-orthodontic treatment, specifically on the anterior teeth, report diminished contentment with the aesthetic quality of their smile and tooth color [3]. Hence, prevention and management of WSLs are of utmost importance. WSLs necessitate a multifactorial approach to management. Preventing demineralization and biofilm formation, in addition to developing techniques to promote remineralization, ought to constitute the initial approach [4]. Fluoride-based approaches are the gold standard in the prevention and management of WSLs. It has been reported, however, that current fluoride therapies are deficient, particularly in cases of caries that have already developed white spots. [5]. Considerable research and utilization of contemporary materials are being tested for the management of WSLs [6]. Particularly, advances in nanotechnology are paving the way for the development of materials that can inhibit biofilm formation as well as remineralize existing enamel decalcifications. Considering the long duration of orthodontic treatment, researchers are focusing on incorporating nanomaterials in orthodontic composites and primers to enhance antimicrobial properties as well as remineralizing properties [7].

Hydroxyapatite nanoparticles (HAPn) have garnered great focus in dental research due to their increased biocompatibility, bioactivity, remineralizing, and antimicrobial effects [8]. A recent study found that by integrating HAPn into dental composites, caries-like enamel lesions could remineralize and release calcium and phosphate ions over an extended period, particularly at potentially cariogenic pH levels [9]. Research conducted in vivo and in vitro has demonstrated that nanohydroxyapatite may remineralize initial enamel lesions with an efficacy comparable to or even surpassing that of fluoride [4], making HAPn a promising material for WSL management. Various metal ions can be added to the structure of HAPn to enhance its antimicrobial properties. In the last decade, metal oxides, especially magnesium oxide nanoparticles (MgNps), have shown potent antimicrobial properties against *Staphylococcus aureus* and *Escherichia coli* in addition to being a stable and biocompatible material [10]. Iron oxide nanoparticles are being extensively researched in dentistry for their ability to eradicate biofilm formation [11]. Hence, the incorporation of these metal ions into the HAPn can enhance the antimicrobial, antibiofilm, and remineralizing properties of the orthodontic composite, thereby combating enamel decalcification. Various metals like silver (Ag), strontium (Sr), copper (Cu), and Cerium (Ce) are being substituted with HAPn for potential antimicrobial properties [12-14] in combating WSLs. Researchers are now developing several combinations of metal ions with HAPn to determine the most effective and biocompatible option. Magnesium (Mg) and iron (Fe) have also demonstrated significant antibacterial properties, as mentioned previously. When coupled with HAPn, these substances may exhibit synergistic effects. Currently, there are no studies that have combined and assessed Fe and Mg co-substituted HAPn in an orthodontic composite to assess its properties. Hence, the present study aims to characterize Fe and Mg co-substituted hydroxyapatite nanoparticles (FeMgHAPn) and assess the antimicrobial properties of FeMgHAPn-incorporated orthodontic composite. The primary objective of the study is to analyze the characteristics of the synthesized FeMgHAPn. The second objective is to evaluate the antimicrobial effect of the FeMgHAPn-infused orthodontic composite on *S. aureus*, *E. coli*, and *Streptococcus mutans* using the agar-well diffusion method.

## Materials and methods

The present study was in vitro and was started after approval from the institutional ethical committee (SRB/SDC/UG-1998/23/ORTHO/014).

Synthesis of the FeMgHAPn

The following steps represent a summary of the synthesis of FeMgHAPn powder using a sol-gel process. Magnesium nitrate hexahydrate (Mg(NO_3_)2•6H_2_O), ferric nitrate nonahydrate (Fe(NO_3_)39H_2_O), diammonium hydrogen phosphate ((NH_4_)_2_HPO_4_), and calcium nitrate tetrahydrate (Ca(NO_3_)2•4H_2_O) were all purchased from Sigma-Aldrich (Merck Group, Darmstadt, Germany) for use in the synthesis process of the FeMgHAPn. Distilled water was used as a solvent. Following the dissolution of 0.486 M Ca(NO_3_)2•4H_2_O, solutions of 0.009 M (Fe(NO_3_)39H_2_O) and 0.005 M Mg(NO_3_)2•6H_2_O were added, respectively. Next, drop by drop, 0.3 M (NH4)2HPO4 solution was added to this mixture. After the new solution changed into a gel, it was combined using a magnetic stirrer and heated to 90°C for three hours. It was then dried for 24 hours at 150°C in an oven. After 2.5 hours of heating, the as-dried material was at 650°C in an electric furnace, and then FeMgHAPn powders were isolated and stored.

Characterization of the FeMgHAPn

The prepared FeMgHAPn powder was characterized using Fourier Transform Infrared Spectroscopy (FTIR), energy-dispersive X-ray analysis (EDX), and scanning electron microscopic (SEM) analysis to study the structure and confirm the composition of the formed nanoparticle.

Incorporation of the FeMgHAPn in orthodontic composite

Following characterization to confirm the successful formation of FeMgHAPn, the nanoparticles were then incorporated into a commercially available orthodontic composite (Transbond XT, Ormco, Orange, CA). The technique for integrating FeMgHAPn was derived from a prior investigation [15]. FeMgHAPn, weighing 0.025 g, was combined with 1 g of orthodontic material using a glass spatula under darkness. Two concentrations, a high concentration of 40 μL and a low concentration of 20 μL, were prepared based on nanoparticle dispersion in the composite done as per previous research [13]. Two dispersion concentrations of the FeMgHAPn in the composite were achieved by diluting 20 and 40 μL of the FeMgHAPn-infused composite in 1 mL of dimethyl sulfoxide (DMSO). The high and low concentration mixes were thereafter introduced into a vortex machine (Labquest, Borosil, Mumbai, India) individually, at a rotation speed of 600 rpm, for a duration of 10 minutes. The nanoparticle composite was thereafter transferred into clean and sealed beakers to shield it from light and avoid dispersion in water. The beakers were coated with black Teflon tape and then subjected to sonication for a duration of 60 to 90 minutes. In the ultrasonication system, water was injected to stabilize the composite temperature by incorporating ice cubes. The FeMgHAPn integrated composite structure was analyzed using SEM analysis. FeMgHAPn was stored in an opaque, airtight container.

Antimicrobial assessment of the FeMgHAPn-incorporated orthodontic composite

The antimicrobial property of FeMgHAPn-incorporated orthodontic composite was assessed using the agar-well diffusion method. The antimicrobial efficacy was tested against gram-positive bacteria *S. aureus* (MTCC-740), *S. mutans* (MTCC-890), and gram-negative bacteria *E. coli* (MTCC-443). The test bacterial cultures of 20 μL, containing 150 cells/mL^-1^, were utilized.

Approximately 100 mL of Mueller Hinton agar was made, sterilized, and placed onto petri plates for the cultivation of *S. mutans*, *S. aureus*, and *E. coli* separately. The plates underwent solidification. Following the process of solidification, the corresponding plates were gently wiped with the bacterial solutions that had been produced beforehand. Following the swabbing process, a gel puncher was used to create three wells on each plate. About 20 μL of FeMgHAPn-incorporated composite was injected into three wells at two different concentrations: 20 and 40 μL. A separate well was filled with a control solution consisting of a combination of streptomycin and amoxicillin antibiotics. The plates were thereafter placed in an incubator set at a temperature of 37°C for a duration of 24 hours. Positive test results were determined by the presence of a zone of inhibition (ZOI) surrounding the well following the incubation time.

## Results

The present in-vitro study was conducted for a period of three months (April-June 2023).

Characterization of the FeMgHAPn

FTIR

**Figure 1:**
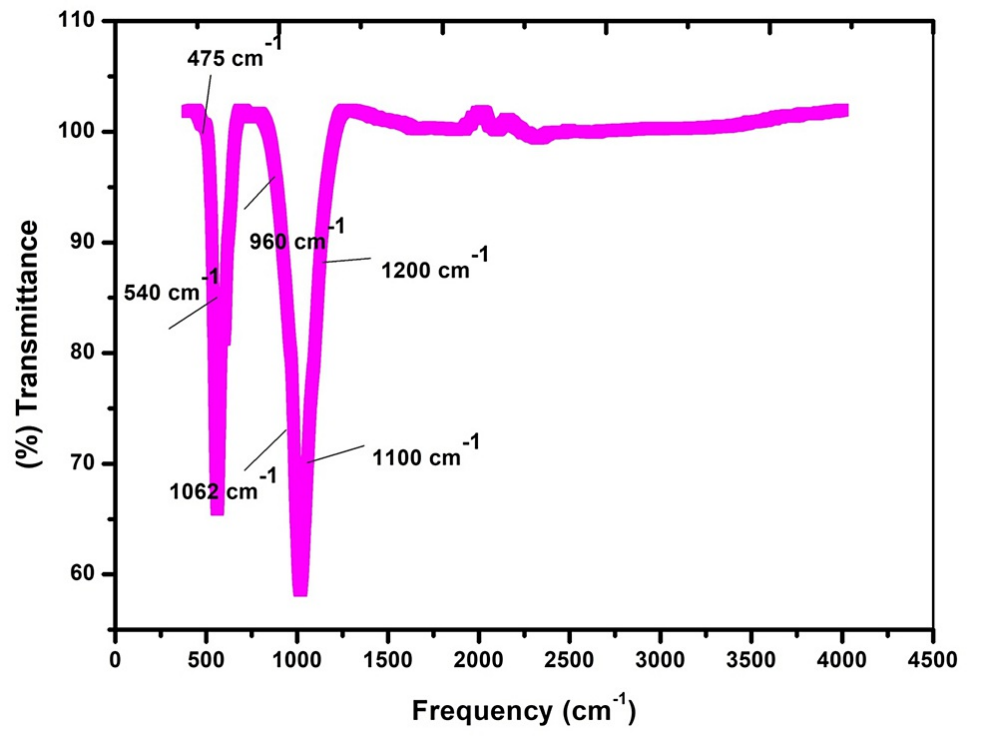
Fourier Transform Infrared Spectroscopy image of the FeMgHAPn powder FeMgHAPn, Fe and Mg co-substituted hydroxyapaptite nanoparticles

Figure 1 illustrates the FTIR spectra of the FeMgHAPn powder. The wave numbers between 1,100 and 1,200 cm^-1^ in the spectrum are attributed to the presence of carbonates, which exhibit measured intensities within this range. The FTIR spectrum exhibited a spectral region spanning from 500 to 650 cm^-1^, which confirmed the presence of bending vibrations of phosphorous (P) and oxygen (O) linked with the phosphate group. The peak band at 475 cm^-1^ in the spectrograph is identified as the doubly degenerate υ2 bending mode of the O-P-O, whereas the smaller peak about 960 cm^-1^ corresponds to the nondegenerate P-O symmetric stretching mode, known as the υ1 mode. The intense band (υ3) corresponding to the PO43-ion is referred to as the P-O stretching vibration mode, and it is situated at a wavenumber of 1,062 cm^-1^. The FeMgHAPn did not exhibit any structural alterations due to the low concentration of cationic substitutions. Nevertheless, the spectrum clearly exhibited all the distinct vibration bands associated with a HAPn material.

EDX

**Figure 2:**
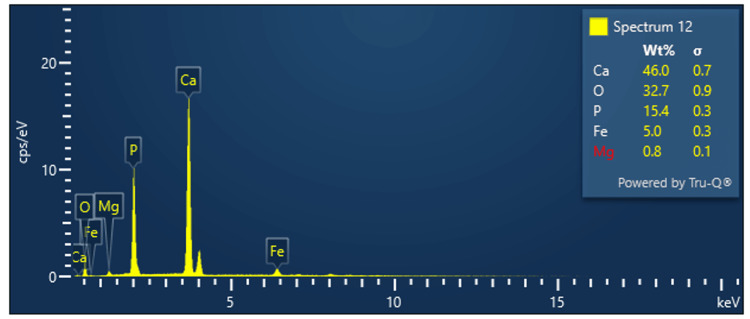
Energy dispersive X-ray spectroscopy image of the FeMgHAPn powder FeMgHAPn, Fe and Mg co-substituted hydroxyapaptite nanoparticles

Figure 2 depicts the EDX spectra of the synthesized FeMgHAPn powder. EDX analysis confirms the presence of Ca, P, O, Fe, and Mg in the sample, and the elements have been distributed in the entire surface of the sample.

SEM

**Figure 3:**
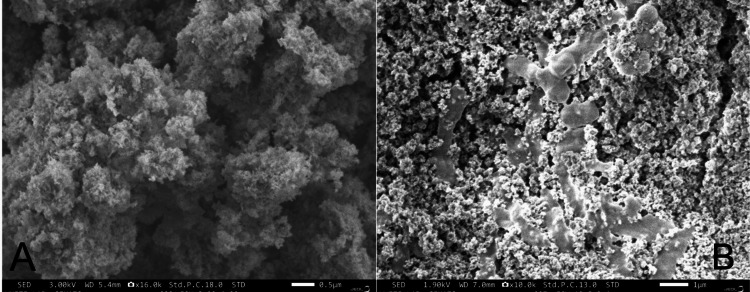
(A) Scanning electron microscopic image of the FeMgHAPn powder; (B) scanning electron microscopic image of the FeMgHAPn-incorporated orthodontic composite FeMgHAPn, Fe and Mg co-substituted hydroxyapaptite nanoparticles

The SEM image of the FeMgHAPn powder and the FeMgHAPn-incorporated orthodontic composite is depicted in Figure 3. The FeMgHAPn powder exhibited a shape characterized by the formation of flaky, layered aggregates. The FeMgHAPn-incorporated orthodontic composite had a morphology that was both flatter and characterized by small, agglomerated structures. The incorporation of nanoparticles into an orthodontic composite resulted in a subtle reduction in the flaky and aggregated structure of the sample's surface morphology. The SEM photos revealed a uniform and layered structure, with subtle variations between the orthodontic composite containing nanomaterials and the nanoparticles alone.

Antimicrobial properties of FeMgHAPn-incorporated orthodontic composite

**Figure 4:**
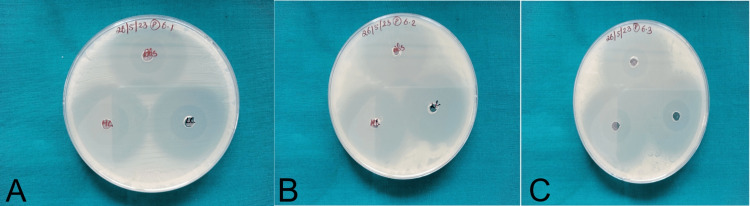
(A) Zones of inhibition against Staphylococcus aureus; (B) zones of inhibition against Streptococcus mutans; (C) zones of inhibition against Escherichia coli

The ZOI measured for all three microbes tested has been indicated in Table 1 and Figure 4. The maximum ZOI for *S. aureus* was seen in the group with a high concentration (28 mm) of FeMgHAPn, followed by a low concentration (25 mm) and the control group (22 mm). For *S. mutans*, the highest ZOI was seen in the high-concentration group (31 mm), closely followed by the low-concentration group (30 mm) and control group (24mm). The maximum ZOI for *E. coli* was seen in the high-concentration group (32 mm), followed by low-concentration (30 mm) and the control group (22 mm).

**Table 1:** Zones of inhibition in mm against three bacteria in the three test groups

Bacteria	Zones of inhibition (mm)		
	Control	Low concentration	High concentration
Staphylococcus aureus	22	25	28
Streptococcus mutans	24	30	31
Escherichia coli	22	30	32

## Discussion

The study synthesized FeMgHAPn, characterized the particles, integrated them into an orthodontic composite, and evaluated their antibacterial properties. The initial investigation revealed that the high concentration (40 μL) of FeMgHAPn resulted in the greatest ZOI against all microorganisms. This work is the first to co-substitute both Fe and Mg with hydroxyapatite nanoparticles (HAPn) and incorporate it in an orthodontic composite to evaluate its antibacterial characteristics. Rangrazi et al. [16] previously utilized MgNps in orthodontic composite to evaluate their antimicrobial effectiveness against *S. mutans*. The study determined that a 1% concentration of MgNps is the minimum concentration required for maximum antibacterial efficiency against *S. mutans*. Predoi et al. [17] conducted a study where they synthesized Mg-doped HAPn using the co-precipitation method and evaluated its antibacterial properties against *Pseudomonas aeruginosa*, *S. aureus*, and *Candida albicans*. The study discovered effective prevention of biofilm formation even at low concentrations (0.009 mg/mL).

Habib et al. [18] conducted a study where antibacterial metal ions, such as Fe, aluminium (Al), zinc (Zn), and nickel (Ni), were added to HAPn to test their effectiveness against a variety of gram-positive and gram-negative bacteria. The investigation revealed that Fe ion-doped HAPn exhibited a significant inhibitory zone of 16 mm, demonstrating the highest effectiveness compared to other investigated ions against *E. coli* and *Salmonella typhi* bacterial strains. The present study also found significant zones of inhibition against *S. aureus* and *S. mutans*, and the inhibition increased with the concentration of the nanoparticle. The antimicrobial properties can be attributed to the Fe and Mg ions, as previous studies have shown that HAPn alone shows poor antimicrobial effects [19]. The present study did not compare the antimicrobial properties of isolated HAPn with FeMgHAPn. Further studies can be done to ascertain the primary contributor to the effective antimicrobial properties.

The current study analyzed the prepared nanoparticle using FTIR, EDAX, and SEM. FTIR is a prominent analytical technique used to study the chemical composition of materials and their interaction with biological systems. Infrared (IR) spectroscopy enables the identification of chemical components in multi-material systems by analyzing their distinctive absorption signatures, known as molecular fingerprints, in the mid-infrared region. This region includes wavelengths ranging from 2.5 to 20 µm. Furthermore, infrared spectroscopy possesses inherent characteristics of rapidity, non-invasiveness, and non-destructiveness [20, 21].

The FTIR examination of the produced nanoparticle exhibited absorption signals that corresponded to hydroxyapatite groups. The addition of metal ions (Fe and Mg) did not cause any changes to the structure of the HAPn. The EDX spectra confirmed the successful inclusion of the metal ions in the composite. The study's strengths lie in its thorough evaluation of the synthesized material's structure through the use of FTIR, EDAX, and SEM techniques both before and after its integration into the composite. The present study used the agar-well diffusion technique to assess the antimicrobial properties of the prepared nanoparticle. It is a widely used technique to assess the bacterial ZOI. Though this technique is useful in identifying antagonism to the test microbes, the minimum inhibitory concentration (MIC) cannot be ascertained. The broth dilution technique can be done additionally to investigate the MIC of the tested nanoparticle [22].

Limitations

The study limitation is attributed to the preliminary nature and the in-vitro design of the investigation. Increasing the sample size and conducting statistical analysis can enhance the study's generalizability. The current study assessed the antimicrobial impact on only three bacteria. The antimicrobial effect of the nanoparticle must be tested against other common oral pathogens. Also, the study did not assess the duration of the material's antibacterial efficacy and the ability to inhibit biofilm formation. The duration of antimicrobial activity is particularly important as orthodontic treatment goes on for a longer duration. The overall performance of the nanoparticle in a clinical setting for a prolonged duration will determine its clinical applicability. The present study establishes a potential combination, which has a long way to go before it can be utilized clinically.

Though the study emphasizes the potential of using this nanoparticle combination to tackle WSL, further research is needed to investigate the material's potential changes in adhesive strength, durability, or wear resistance due to nanoparticle incorporation. This can shed valuable insights into the material's overall performance in clinical settings.

## Conclusions

The present study synthesized FeMgHAPn-incorporated orthodontic composite and found a potential antimicrobial effect against *S. mutans*, *S. aureus*, and *E. coli*. The antimicrobial effect increased with the increase in the concentration of the nanoparticle. The study sets the stage for future research using the FeMgHAPn integrated composite for combating WSLs.
